# Supplementary material for: The Effect of Bimanual Intensive Functional Training on Somatosensory Hand Function in Children with Unilateral Spastic Cerebral Palsy: An Observational Study
Source: J Clin Med. 2023 Feb 17;12(4):1595. doi: 10.3390/jcm12041595 (PMC9960591; doi:10.3390/jcm12041595)
Supplement: Supplementary file 1 [file jcm-12-01595-s001.zip › Supplementary file S1 TIDieR 230112.pdf]

Supplementary information to our paper, titled: "The effect of bimanual intensive functional training on somatosensory hand function in children with unilateral spastic cerebral palsy".

S1 Intervention Description and Replication (TIDieR)

Brief name

BIMT (Bimanual Intensive Movement Therapy) program.

Why: rationale, theory, or goal of the elements essential to the intervention.

Social participation, independence and self-efficacy are restricted in children with CP as they experience limitations in the performance of daily activities. Intensive goal-oriented upper limb therapies have been found to be effective in promoting bimanual performance and daily functioning in children with unilateral CP. This scientific knowledge is translated into a clinical intervention programme by researchers and clinical staff of Adelante Paediatric Rehabilitation Centre in Houthem, the Netherlands. All the staff has over 7 years of experience with working with children with unilateral spastic CP and is specialised in hand function problems in these children. The programme is based on motor learning principles and functional therapy, according to the (inter)national guidelines in CP, i.e. the therapy is goal directed, using a context based approach, aimed at active participation of the child, focusing on activity and participation and incorporating parent involvement.

What: materials and procedures

The programme focuses on using both hands in numerous everyday two-handed skills. The affected hand is considered the assisting hand in a stabilising or supporting role. Potential candidates are extensively screened prior to participation by members of our 'upper limb' expert team. During this screening every participant formulates their own personal treatment goals and needs. Together with the therapist and parents, these goals are ranked into a top three. Performance of these three goals is assessed, and after a task analysis, they are translated into individualised goal-directed therapy sessions, by experienced therapists. Boxes with necessary materials are assembled per participant. All training is performed on site.

Therapy starts from the moment of waking up, when self-care activities such as dressing and grooming are practised in the context. All materials and items used during the day are chosen so as to enhance two-handed operation. Participants are actively involved in the preparation of the meals and cleaning up afterwards. The breakfast session is followed by a therapy block of 90-120 minutes in which the specific personal treatment goals are trained. In the afternoon activities that specifically focus on personal goals are alternated with group activities (survival, sports, bimanual gaming and

recreational activities). Again these activities require the use of two hands, such as, for example, the climbing course. These activities are group based and coordinated by the supervisors or hired experts. Participants receive a daily total of 6-7 hours of intensive therapy, for 15 days, amounting in total of 80 to 90 hours for the entire program. An example of the controlled training is given by the random day schedule, presented in table 1.

Table S1. Example of a random day of the BIMT program

Day	week nr. 1
**Thursday**	**Activity**
08:00-08:45	Breakfast
09:00-09:30	Bimanual crafting
09:30-10:00	Bimanul Powertraining (in line with the personal goals of the participant)
10:00-10:45	Gross motor bimanual activities such as climbing
10:45-11:00	Break
11:00-11:45	Fine motor bimanual activities
11:45-12:30	Preparing dinner
12:30-13:15	Break
13:15-14:45	Goal oriented training
14:45-15:00	Break
15:00-15:30	Bimanual gaming
15:30-16:00	Arts project
16:00-17:00	Various bimanual activities such as baking or crafting
17:00-18:00	Cooking
18:00-20:00	Dinner

Before start of the program, caregivers are invited to a special caregiver meeting. In this meeting, the therapists provide information on the concept of bimanual intensive therapy, the content of the programme and practical information. All participants and their caregivers are introduced to a personal buddy. Seven days after the start of the program, caregivers and siblings are joining the program for two days. During these two days, parents can see and experience their child's development with their own eyes. In addition they become are shared decision makers for the goal

directed training in the second week. The last day of the program the parents are invited for a final consultation. Together, the effects of the programme are evaluated. We also discuss how the skills learned, can be applied permanently in the home environment.

<u>Who provided</u>

In order to encourage training intensity, all participants are paired with a personal buddy who continuously prompts the participant to use both hands when performing activities throughout the day. These buddies are interns in occupational therapy, movement sciences, medicine or sports training. The interns are trained by the therapists in the principles of goal-oriented bimanual intensive training and transfer of the achieved goals to other situations, most importantly the home environment. During the programme the students are supervised by experienced therapists. In terms of manpower, one healthcare professional supervises three participants, in which they are supported by the buddies.

<u>How</u>

The therapy involves hands-off training and the therapists have a coaching role. In this, they are assisted by the buddies. The buddies encourage the participant to use both hands when performing activities throughout the day. They also keep a diary in which they record the time-on-task and in which they take notes on things they notice. These are discussed with the supervising therapist at the end of the afternoon each day. The therapists can be either occupational therapists or physical therapists. More detail on how the therapy is given has already been described in the sections above

<u>Where</u>

The BIMT programme is run every year during the first two weeks of the summer holidays because adolescents from all over the country can participate. During these two weeks, all participants stay on site and sleep in a special unit of the rehabilitation centre. The rehabilitation centre provides facilities such as a gym, a garden with trees as climbing facility and a swimming pool. A maximum of ten participants can be included into the program.

<u>Tailoring</u>

During the initial screening the participant formulates his/her own personal treatment goals and needs. Together with the therapist and caregivers, these goals are ranked into a top three. Performance of these three goals is assessed, and after an extensive task analysis, they are translated into individualised goal-directed therapy sessions, by experienced therapists. Boxes with necessary

materials are assembled per participant. The individualised training of the personal treatment goals is alternated by group activities such as climbing or crafting, but even during these group activities the coaching of the participants is focused on their personal goals.

Modifications

The dosage and schedule of the controlled training is never modified. Possible modifications made, relate to the type of activity. For example, djembé instead of drumming. In the year 2020, the year of the COVID-19 pandemic, precautions had to be taken. Staff and participants were tested prior to participation, mouth masks were worn all day and social distance rules had to be observed.

Actual

The intervention was delivered as planned. The time-on-task diaries indicate an actual therapy dose of 6 to 7 hours per day.
