# Supplementary material for: The Effect of Bimanual Intensive Functional Training on Somatosensory Hand Function in Children with Unilateral Spastic Cerebral Palsy: An Observational Study
Source: J Clin Med. 2023 Feb 17;12(4):1595. doi: 10.3390/jcm12041595 (PMC9960591; doi:10.3390/jcm12041595)
Supplement: Supplementary file 1 [file jcm-12-01595-s001.zip › Supplementary file S2 GAS 230112.pdf]

Supplementary information to our paper, titled: "The effect of bimanual intensive functional training on somatosensory hand function in children with unilateral spastic cerebral palsy".

S2 A list of the individual treatment goals for all participants:

Participant	Most important bimanual treatment goal (GAS score 0)
**1**	Participant is able to put a chair upside down on a table within one minute and ten seconds
**2**	Participant is able to wrap a can of shaving cream within one minute and thirty seconds
**3**	Participant is able to peel an onion and cut it into equally sized cubes within two minutes and thirty seconds
**4**	Participant is able to chop a quarter of a bell pepper with a knife in one minute
**5**	Participant can cut a piece of raw chicken meat in half with a knife and fork with the fork slipping away only one time
**6**	Participant closes a zipper of a cardigan within twenty-eight seconds
**7**	Participant can stack up four chairs on a stack of four chairs within one minute and ten seconds
**8**	Participant cuts half of a bell pepper with a knife in fifteen pieces within two minutes and fourteen seconds
**9**	Participant is able to put a watch strap in the buckle of the watch, and strap the watch within fifty-six seconds
**10**	Participant is able to tie her own hair into a simple pony tail by grasping the hair with the left hand and fixing the pony tail using a hair tie with the right hand
**11**	Participant is able to tie his shoes into a loose knot in five steps
**12**	Participant is able to dry a cup by holding a dish cloth in the left hand and the cup in the right hand, within two minutes
**13**	Participant is able to peal one side a medium-sized potato using a vegetable peeler in one minute and fifteen seconds, by fixing the potato with a fork in the left hand
**14**	Participant is able to cut a raw piece of meat in nine slices in one minute and thirty seconds
**15**	Participant is able to cut half a carrot into 5mm slices in one minute
**16**	Participant is able to peal one side of a medium-sized potato using a vegetable peeler in one minute, with the potato slipping away maximum two times
**17**	Participant is able to transport garbage on a surface of 4x2 square metre without dropping any garbage in three minutes

**18**	Participant is able to type ninety characters on a computer keyboard in one minute by using both hands
**19**	Participant is able to tie her own hair into a simple pony tail by wrapping the hair tie around the ponytail two times
**20**	Participant is able to tie her own hair into a tight ponytail by wrapping the hair tie around the ponytail three times
**21**	Participant is able to type sixty characters on a computer keyboard in one minute and ten seconds by using two fingers of the left hand
**22**	Participant is able to peel a pear by using a vegetable peeler in one minute
**23**	Participant is able to peel a medium-sized potato using a vegetable peeler in one minute and thirty seconds
**24**	Participant is able to peel the plastic packaging off one-third of a sausage and cut this piece into small pieces within one minute and thirty seconds
